# Whole-Genome Sequence of *Coxiella burnetii* Nine Mile RSA439 (Phase II, Clone 4), a Laboratory Workhorse Strain

**DOI:** 10.1128/genomeA.00471-17

**Published:** 2017-06-08

**Authors:** Jess A. Millar, Paul A. Beare, Abraham S. Moses, Craig A. Martens, Robert A. Heinzen, Rahul Raghavan

**Affiliations:** a Department of Biology and Center for Life in Extreme Environments, Portland State University, Portland, Oregon, USA; b *Coxiella* Pathogenesis Section, Laboratory of Bacteriology, Rocky Mountain Laboratories, National Institutes of Health, Hamilton, Montana, USA; c Genomics Unit, Research Technologies Section, Rocky Mountain Laboratories, National Institutes of Health, Hamilton, Montana, USA

## Abstract

Here, we report the whole-genome sequence of *Coxiella burnetii* Nine Mile RSA439 (phase II, clone 4), a laboratory strain used extensively to investigate the biology of this intracellular bacterial pathogen. The genome consists of a 1.97-Mb chromosome and a 37.32-kb plasmid.

## GENOME ANNOUNCEMENT

*Coxiella burnetii* is a Gram-negative intracellular bacterium that causes an influenza-like illness in humans called Q fever (1). Most infections occur through inhalation of aerosols originating from domestic livestock operations. Within the host cell, the pathogen becomes metabolically activated upon delivery into an acidic lysosome-like vacuole (2). The only *C. burnetii* virulence factor established in an immunocompetent animal model of infection is full-length lipopolysaccharide (LPS), which is synthesized by virulent phase I bacteria (3). Upon serial *in vitro* passage, phase I bacteria convert to avirulent phase II bacteria, which produce truncated LPS lacking O antigen and several core sugars (4–8).

The Nine Mile RSA439 (phase II, clone 4) strain (NMII) was derived from the Nine Mile strain, which was originally isolated in 1935 from the tick *Dermacentor andersoni* in Montana (9). The Nine Mile strain was passaged 94 times in embryonated hen’s eggs and then plaque purified to generate NMII (10, 11). NMII has an ~26-kb chromosomal deletion that eliminates several LPS biosynthetic genes and is associated with the production of a severely truncated LPS (12–16). Because of clonality, avirulence in a guinea pig model of infection, and lack of phase reversion, NMII is considered a biosafety level 2 (BSL-2) bacterium (3, 17, 18). Other *C. burnetii* strains are considered BSL-3 bacteria and are regulated as select agents by the U.S. Centers for Disease Control and Prevention (18).

The NMII genome has not yet been sequenced; consequently, most researchers use the published genome of the Nine Mile RSA493 phase I strain (NMI) for reference (19). This occasionally leads to inconclusive results; for instance, the gene *caeA* is not annotated as a functional protein-coding gene in the NMI genome, but transcriptome analysis of NMII indicates its presence (20, 21). Thus, a fully annotated genome of the widely used NMII laboratory strain is needed to better understand the unique biology of this intracellular pathogen.

NMII was grown in ACCM-2 at 37°C in a 2.5% O_2_/5% CO_2_ environment in a tri-gas incubator (New Brunswick Scientific, NJ), as described previously (22). DNA was isolated from a 500-ml 7-day culture using phenol-chloroform with gentle cell disruption using a vortex adapter (Qiagen, CA) in order to minimize DNA fragmentation. DNA was sequenced using the PacBio RS II platform (Pacific Biosciences, USA), which generated a library containing 86,731 reads with an average length of 7,565 bp. Reads were assembled using HGAP 2.3.0 (23), which returned three contigs. The two chromosome contigs and the plasmid contig were closed in SSPACE 2.0 (24) using trimmed Illumina MiSeq 75-bp paired-end reads (5.50 million). Finally, CLC Genomics Workbench 6.5 (Qiagen) was used to map all Illumina and PacBio reads to the NMII chromosome (410× and 290× coverage, respectively) and plasmid (240× and 30× coverage, respectively) scaffolds to generate the consensus genome sequence. As expected, relative to the genome of NMI, homologs of genes CBU_0679 to CBU_0697 were completely deleted from the NMII genome (3, 13–16). In addition, partial deletions of homologs of CBU_0678, CBU_0698, and CBU_0918 and several single nucleotide polymorphisms were observed.

### Accession number(s).

The complete genome sequence of *C. burnetii* Nine Mile RSA439 (phase II, clone 4) has been deposited in GenBank under the accession numbers CP020616 (chromosome) and CP020617 (plasmid).
